# Parental Cognition Regarding Children’s Sugar Consumption and Its Implications for Dental Caries Prevention: A Systematic Review

**DOI:** 10.3390/children13091236

**Published:** 2026-09-12

**Authors:** Areena Tahir, Esther García-Miralles, Juan Ignacio Aura-Tormos, Clara Guinot-Barona, Laura Marqués-Martínez

**Affiliations:** 1 Department of Stomatology, Faculty of Medicine and Dentistry, Universitat de València, 46010 Valencia, Spain; areena.tahir@uv.es (A.T.); m.esther.garcia@uv.es (E.G.-M.); juan.aura@uv.es (J.I.A.-T.); laura.marques@ucv.es (L.M.-M.); 2 Dentistry Department, Medicine and Health Faculty, Catholic University of Valencia, 46001 Valencia, Spain

**Keywords:** parental cognition, parental perception, sugar intake, sugar-sweetened beverages, behavioural modelling, food environment, dental caries, children, nutritional literacy, health literacy, systematic review

## Abstract

Background/Objectives: Parental perceptions, beliefs, attitudes, and knowledge may influence children’s sugar exposure, but these cognitive constructs are distinct from children’s actual intake. This review synthesised parental cognition regarding children’s sugar consumption and secondarily examined its relationship with reported or measured intake and implications for caries prevention. Methods: PubMed, Scopus, PsycINFO, CINAHL, and Web of Science were searched for primary studies published from January 2016 to April 2026. Study selection and narrative synthesis followed PRISMA 2020. Results: We included 35 studies from ten countries: 19 cross-sectional, 6 qualitative, 4 experimental/randomised, 2 pre-post, 1 longitudinal, and 3 other survey-based or mixed-design studies. Misperceptions of product healthfulness and limited knowledge of sugar content were common. Associations between cognition and children’s intake were inconsistent; parental modelling, household availability, cultural norms, and commercial influences were also associated with sugar exposure. Most evidence was observational, measures were heterogeneous, and no study measured dental caries directly. Conclusions: Parental cognition is relevant but insufficient on its own to explain children’s sugar exposure. Caries-preventive counselling should combine factual education with attention to caregiver behaviour and the home food environment.

## 1. Introduction

Dental caries remains the most prevalent chronic disease worldwide and one of the most common non-communicable diseases in childhood. Untreated caries affects approximately 2.4 billion individuals globally, primarily in permanent dentition, while early childhood caries (ECC) affects hundreds of millions of children in primary dentition worldwide [1]. The World Health Organization (WHO) recommends limiting free sugar consumption to less than 10% of total energy intake, with additional benefits below 5% (approximately 25 g/day) [2]. Despite these recommendations, epidemiological data consistently demonstrate that children’s free sugar intake substantially exceeds these thresholds globally, largely driven by SSBs, ultra-processed foods, and sweetened dairy products [3,4]. Its development is influenced by biological, behavioural, psychosocial, and environmental factors, among which dietary free-sugar exposure is one of the principal modifiable determinants.

Parents constitute the primary architects of the early-life food environment. Parental practices encompassing food availability in the home, modelling of eating behaviours, active dietary education, and feeding rules are significantly associated with children’s SSB and high-sugar food consumption [5,6]. Crucially, the effectiveness of these practices depends not only on parental behaviour per se, but also on how caregivers perceive their children’s diet: if parents fail to recognise excess sugar intake, preventive action is unlikely to be initiated.

Multiple studies indicate that parents tend to underestimate the sugar content of foods and beverages consumed by their children and often misclassify cariogenic products—such as commercial fruit juices, sports drinks, and flavoured waters—as healthy choices [7,8]. Furthermore, the gap between knowledge and practice has been documented: many caregivers who acknowledge the harms of sugar do not translate this awareness into effective dietary control [9], raising the possibility that parental cognition alone—however accurate—may be an incomplete explanatory framework for children’s actual sugar exposure, and that behavioural and environmental factors warrant consideration alongside it. These dynamics are relevant to dental caries prevention primarily through their influence on children’s sugar exposure, the principal modifiable driver of the cariogenic process: frequent and prolonged exposure of tooth surfaces to fermentable carbohydrates drives demineralisation and disrupts the ecologically balanced oral microbiome towards a cariogenic state [10,11].

Mechanistically, excessive sugar consumption promotes a dysbiotic, acidogenic oral biofilm associated with progressive enamel demineralisation [12,13] (examined further in the Discussion). Since parents largely determine children’s dietary habits during the first years of life, their perceptions, beliefs, knowledge, and feeding practices may indirectly influence this process.

Although individual studies have examined different aspects of parental cognition regarding children’s sugar consumption, an up-to-date systematic synthesis integrating parental perceptions, beliefs, attitudes and knowledge with children’s reported or measured sugar intake, in the context of dental caries prevention, is lacking. The primary objective of this systematic review was therefore to synthesise the available evidence on parental perceptions, beliefs, attitudes, and knowledge regarding children’s sugar consumption. A secondary objective was to examine how these cognitive constructs relate to children’s reported or measured sugar intake and to consider the implications of both for dental caries prevention.

The review framework was structured using PICo as follows: Population—parents and primary caregivers of children and adolescents aged 0–18 years; Phenomenon of Interest—parental perceptions, beliefs, attitudes, and knowledge regarding children’s sugar or SSB consumption; and Context—children’s dietary sugar exposure and dental caries prevention. The primary review question concerned parental cognition itself. A distinct secondary question examined whether and how these cognitive constructs were associated with children’s reported or measured sugar intake; direct measurement of child intake was therefore not required for study eligibility.

## 2. Materials and Methods

This systematic review was conducted and reported in accordance with the Preferred Reporting Items for Systematic Reviews and Meta-Analyses (PRISMA 2020) statement [14].

### 2.1. Eligibility Criteria

Inclusion criteria were as follows: (1) primary empirical studies whose topic included parental or caregiver cognition—operationalised as perception, belief, attitude, or knowledge—regarding sugar or SSB consumption in children or adolescents; (2) parents, mothers, fathers, or primary caregivers of children or adolescents aged 0–18 years, or an independently extractable subgroup within that age range; (3) quantitative, qualitative, or mixed-methods primary research; (4) full text available in English or Spanish; (5) peer-reviewed journal publication; and (6) publication between January 2016 and April 2026. Children’s reported or measured sugar intake was treated as a separate secondary outcome and was not required for eligibility.

Exclusion criteria were as follows: (1) no sugar- or SSB-specific parental cognition outcome; (2) exclusive focus on non-nutritive sweeteners without reference to sugar; (3) parents included only as informants, without a reported perception, belief, attitude, or knowledge outcome; (4) reviews, qualitative evidence syntheses, editorials, letters, protocols, and other non-primary reports; (5) populations outside the 0–18-year criterion without separately extractable eligible data; and (6) duplicate or secondary reports of an already represented cohort.

Primary and secondary outcomes: the primary outcome of this review was parental or caregiver cognition—defined as perceptions, beliefs, attitudes, and knowledge—regarding children’s sugar consumption, as measured and reported in the included studies. Secondary outcomes were as follows: (i) the relationship between parental cognition and children’s reported or measured sugar intake; (ii) determinants of parental knowledge and nutritional literacy; (iii) barriers and facilitators of parental sugar provision; and (iv) implications for dental caries risk and prevention. Parental cognition and actual child sugar consumption were thus treated as methodologically distinct constructs: every included study was required to address the primary outcome, whereas reporting a direct or objective measure of child intake was not an eligibility requirement.

### 2.2. Information Sources and Search Strategy

An initial exploratory search of PubMed and Scopus was conducted on 2 April 2026. After the first peer-review round, the eligibility framework and outcome hierarchy were clarified, and the search was expanded. On 24 April 2026, PubMed and Scopus were re-searched, and PsycINFO, CINAHL, and Web of Science Core Collection were searched for records published from January 2016 to April 2026. Records from the five searches conducted on 24 April were combined and deduplicated, and the final screening and synthesis reported here were based on that unified record set. The January 2016 lower limit defined an approximately decade-long contemporary evidence window beginning immediately after publication of the WHO 2015 sugar-intake guideline [2]. This window was selected to prioritise evidence generated within the subsequent food-labelling, beverage-marketing, and public-health context; it was not intended to imply that earlier studies were irrelevant. The restriction remains pragmatic and may have excluded relevant pre-2016 evidence. Search syntax and fields were adapted to each platform. Consequently, retrieval volumes are not directly comparable: Scopus was searched in TITLE-ABS-KEY and restricted to English or Spanish articles, Web of Science used Topic fields without a language filter, and the EBSCOhost searches used TX and controlled-vocabulary headings. The marked yield difference between Scopus and Web of Science should therefore not be attributed solely to database coverage. During strategy development, “added sugar*” and “soda” were considered as independent terms; pilot searches indicated substantial overlap with records already captured by “sugar-sweetened beverage*” and “sugary drink*”. Broader beverage terms without an explicit sugar qualifier, such as “soft drink*”, were not added independently in order to preserve the review’s focus on sugar-specific parental cognition rather than general beverage intake. Nevertheless, omission of additional independent synonyms may have reduced sensitivity. Complete verbatim strategies, operators, field codes, limits, yields, exact dates, and the rationale for term selection are reported in Table S1.

### 2.3. Study Selection

All records identified across the five databases on 24 April 2026 (*n* = 1361) were exported into a single reference-management library and deduplicated together, removing 292 duplicate or overlapping records and yielding 1069 unique records for title and abstract screening. Two independent reviewers (A.T., E.G.M.) screened all 1069 records against the finalised eligibility criteria (Section 2.1); disagreements were resolved by consensus. A total of 934 records were excluded on the basis of title and abstract, and 135 reports were sought for full-text retrieval. Six reports could not be retrieved despite a targeted search [15,16,17,18,19,20]), leaving 129 reports that were retrieved and assessed for eligibility.

**Figure 1:**
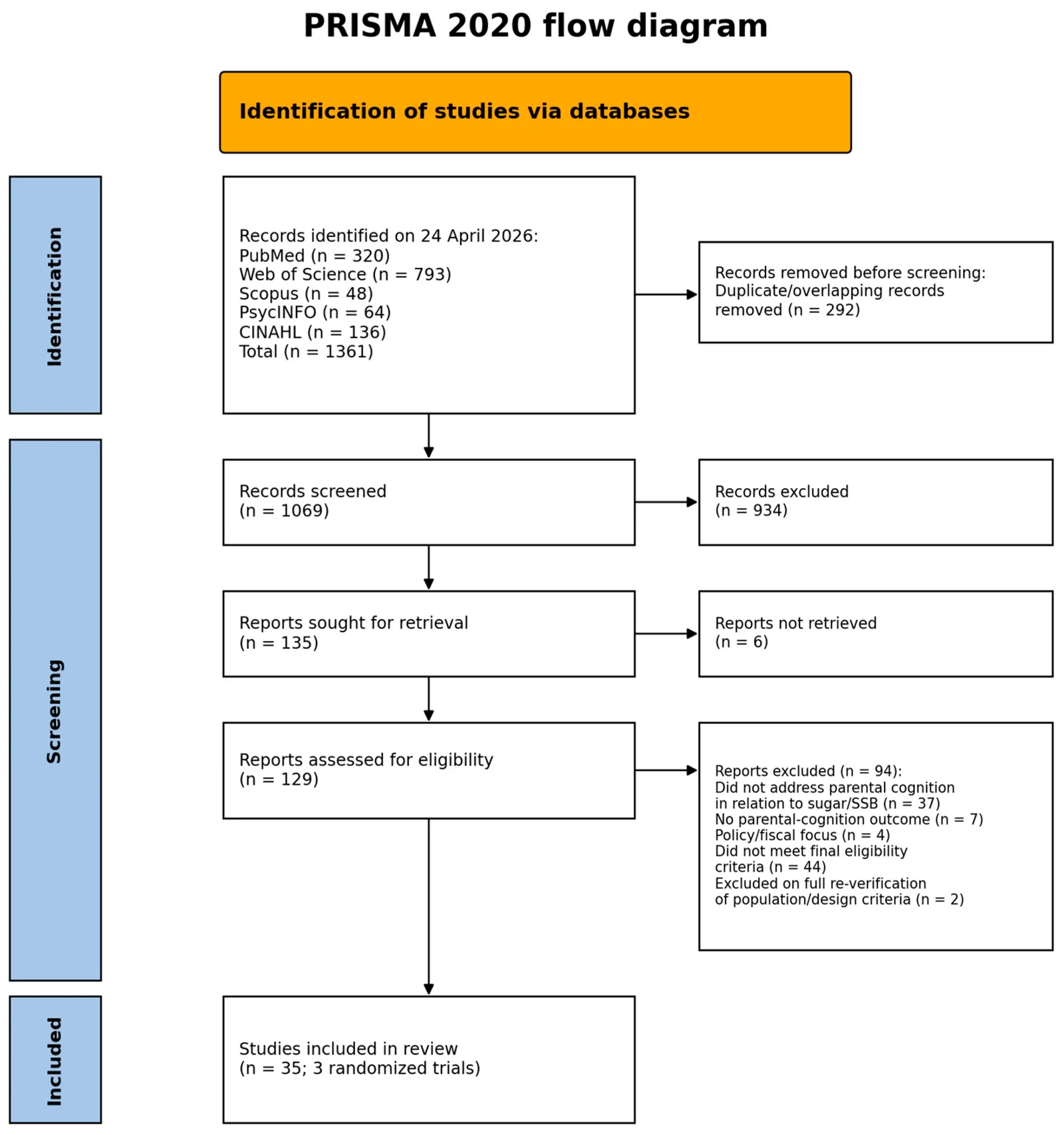
PRISMA 2020 flow diagram for the systematic review, based on the unified five-database search (PubMed, Scopus, PsycINFO, CINAHL, and Web of Science Core Collection) completed on 24 April 2026.

Ninety-four of the 129 retrieved reports were excluded after full-text assessment: 37 did not address parental cognition specifically in relation to sugar or SSB consumption; 7 did not report a parental perception, belief, attitude, or knowledge outcome; 4 addressed policy or fiscal measures without an eligible parental-cognition outcome; 44 failed another prespecified eligibility criterion, including general dietary or feeding studies without an extractable sugar-specific cognition outcome and secondary reports of an already represented cohort; and 2 were near-miss exclusions identified during re-verification. Qiu et al. included participants aged 10–20 years without separately extractable data for those aged 18 years or younger [21], and Killion et al. was a thematic synthesis of previously published studies rather than a primary caregiver study [22]. The remaining 35 reports met the final eligibility criteria. The full selection process is shown in Figure 1.

### 2.4. Data Extraction

Two reviewers (A.T. and E.G.M.) independently extracted data using a pre-piloted form. Variables included first author, year, country, design, sample size, child age, parental construct, assessment method, child dietary outcome where reported, and findings relevant to the PICo question. Disagreements were resolved by discussion, with a third author (L.M.-M.) consulted when necessary. Construct and outcome categories are defined in Table 1.

### 2.5. Risk of Bias Assessment

Risk of bias was assessed with design-appropriate instruments: the JBI critical-appraisal checklists for analytical cross-sectional, qualitative, quasi-experimental, and cohort studies [54], and Cochrane RoB 2 for randomised experiments [55]. Bestle et al. and Cui et al. were assessed with the unmodified 11-item JBI cohort checklist because both used longitudinal repeated measurements. Because JBI and RoB 2 differ fundamentally in structure and judgement rules, no common numerical score or cross-tool Low/Moderate/High scale was applied. Official RoB 2 overall judgements were retained; JBI responses are presented item by item without conversion into review-defined global categories.

Risk-of-bias assessment was performed independently by two reviewers (A.T. and E.G.M.), with disagreements resolved by discussion and consultation with L.M.-M. when required. For this revision, every judgement was re-checked against the complete study report. Table S3 presents the resulting item- or domain-level judgements and an explanation for each study. Y, N, U, and NA denote yes, no, unclear, and not applicable for JBI instruments; RoB 2 retains its official domain and overall terminology. No numerical score, universal cut-off, or cross-tool global category was applied. Appraisals informed the cautious narrative interpretation but were not used to exclude studies or rank predictors.

### 2.6. Data Synthesis

Because designs, parental-cognition constructs, and dietary measures were heterogeneous, findings were synthesised narratively, and no meta-analysis was attempted. Four domains were defined for the revised synthesis: (i) parental perception and knowledge; (ii) relationships between parental cognition and children’s reported or measured sugar intake; (iii) behavioural, family, and environmental correlates of sugar exposure; and (iv) implications for dental caries prevention. Two reviewers (A.T. and E.G.M.) independently assigned findings to one or more domains and resolved discrepancies through discussion, consulting L.M.-M. when necessary. Within domains, studies were grouped by design. Comparative language was restricted to differences in the observed consistency of associations and did not treat cross-sectional associations as causal effects or use post hoc global quality rankings.

## 3. Results

### 3.1. Study Characteristics

The 35 studies included in this review were published between 2016 and 2025; although the search extended to April 2026, no eligible study published in 2026 met the inclusion criteria. The evidence reflects a marked increase in research on parental perceptions, beliefs, and knowledge regarding children’s sugar consumption over the past decade. Studies were conducted across ten countries, with a strong concentration in the United States (*n* = 21, 60%), followed by China (*n* = 2), and two studies each from Denmark, the Netherlands, Lebanon, and Australia; the remaining countries—Portugal, Singapore, Germany, and the United Kingdom—contributed one study each. Findings are reported below in two analytically distinct groups: parental cognition itself, comprising perception and knowledge (Section 3.3 and Section 3.4), and its relationship to children’s actual sugar intake and to behavioural, family, and environmental determinants (Section 3.5 and Section 3.6).

Cross-sectional designs predominated (*n* = 19, 54%), followed by qualitative studies using interviews or focus groups (*n* = 6, 17%). The remaining studies comprised four experimental or randomised designs, two uncontrolled pre-post evaluations, one two-wave longitudinal panel, and three other survey-based or mixed designs. Sample sizes ranged from small qualitative samples to cohorts exceeding 3000 participants.

Regarding the parental construct assessed, knowledge was the most common single focus (*n* = 9), followed by perception and belief (*n* = 7 each); five studies assessed attitude specifically, six assessed more than one construct simultaneously, and one assessed provision practices (Table 1). Children’s dietary outcomes, when measured, most often related to sugar-sweetened beverage intake or total sugar intake (*n* = 9 each); notably, 14 studies (40%) did not include a direct child dietary outcome, relying instead on parental reports of provision practices or perceptions.

### 3.2. Risk of Bias

Design-specific appraisal information is reported in Table 2 and item by item in Table S3. Official RoB 2 judgements were retained for four randomised experiments. For studies appraised with JBI, item-level responses are shown without conversion into a common Low/Moderate/High category, because the instruments are not directly interchangeable and no validated cross-tool conversion rule exists.

Across instruments, recurring limitations included absent or incompletely reported psychometric validation of exposure or outcome measures, reliance on self-reported dietary behaviour, incomplete control of confounding, lack of a concurrent control group in the two uncontrolled pre-post evaluations, and limited reflexive reporting in several qualitative studies. These concerns were used to qualify the narrative interpretation but not to produce pooled percentages, rank studies, or claim that one predictor was methodologically “stronger” than another. The complete item-level matrix and study-specific justifications are provided in Table S3.

### 3.3. Parental Perceptions of Children’s Sugar Consumption

Having established the overall characteristics and methodological quality of the included evidence base (Section 3.1 and Section 3.2), we turn first to what parents and caregivers perceive about their children’s sugar consumption. Nine studies examined parental—or, in two cases, child—perceptions of the healthfulness of foods and beverages containing added sugar. Across designs and countries, a consistent pattern emerged: parents and caregivers systematically misjudged which products were healthy, typically overestimating the healthfulness of sugar-containing foods and beverages relative to their actual nutritional content.

This misjudgement was evident at the level of everyday beverage choices: a substantial proportion of parents believed specific sugar-sweetened beverages were healthy despite their high sugar content [8]. Specific product attributes were associated with this misperception: descriptors such as ‘natural’ or ‘made with fruit’ were linked to a ‘health halo’ and to parents judging beverages as healthier than they were, independent of actual sugar content [36,46]. In one qualitative study, this health halo extended to normalising daily sugary drink consumption altogether, with parents showing limited awareness of the actual sugar content of everyday beverages [36].

Perceptions also varied in relation to product labelling and marketing claims rather than objective nutritional content. Parents who perceived a product as healthier, or who responded to specific claims such as ‘vitamin C’ or packaging suggesting a more wholesome product, provided sweetened drinks to their children more frequently; conversely, claims emphasising reduced or no added sugar were associated with less frequent provision, indicating that label-driven perception, rather than actual composition, was associated with provision decisions in either direction [41].

A distinct but related pattern concerned discrepancies between parental perception and objectively measured behaviour or intake. Parents overestimated the overall healthfulness of their children’s diets relative to their children’s actual sugar intake [34]. Similarly, in a large dyadic study, children perceived flavoured milk and juice as considerably healthier than they actually were, yet parents’ own consumption behaviour, not their perception, was associated with their child’s intake, suggesting that perception and behavioural modelling may represent partly distinct correlates [49]. A comparable dissociation was observed for sports drinks: caregivers’ belief that these drinks were ‘healthy’ or ‘necessary for hydration’ was more strongly associated with adolescent consumption than adolescents’ own attitudes were [24]. These perception-intake gaps are examined further, against objectively verified dietary data, in Section 3.5.

One qualitative study additionally highlighted an apparent paradox in parental risk perception: several parents actively preferred offering sugar over non-nutritive sweeteners to their children, a preference that participants attributed to concern about the safety of artificial sweeteners rather than concern about sugar itself [32], a finding that illustrates the association of perceived risk, not only perceived healthfulness, with parental sugar-related decisions.

Overall, this body of evidence indicates that parental misperception of sugar content and healthfulness is widespread, consistent across qualitative and quantitative designs, and associated with a recurring set of factors: naturalistic or health-related label claims, low salience of sugar content relative to other product attributes, and, in some cases, perceived risks associated with alternatives to sugar.

### 3.4. Knowledge and Nutritional Literacy

Beyond perception, a related but conceptually distinct question is whether parents possess accurate factual knowledge about sugar and dietary guidelines: Section 3.3 concerned how healthy parents judged a product to be, whereas this section concerns whether parents could correctly answer specific, verifiable questions about sugar content or recommended intake. Thirteen studies assessed parental—or, in one case, child—knowledge and nutritional literacy regarding sugar, spanning baseline knowledge levels, the influence of labelling on knowledge accuracy, and the effects of educational interventions.

Baseline knowledge about sugar recommendations and content was consistently limited. Only 14% of parents attending a paediatric clinic could correctly answer three questions about American Academy of Pediatrics juice guidelines [29] (its knowledge-intake association is examined further in Section 3.5), and caregivers of paediatric patients more broadly reported limited knowledge and inconsistent perceived control over their child’s sugar and sugar-sweetened beverage intake [31]. Knowledge was also found to be nuanced by sugar type: parental knowledge about added sugar was associated with higher, not lower, dairy beverage consumption, and was unrelated to sugar-sweetened beverage intake specifically, indicating that general nutrition knowledge does not translate uniformly across product categories [6]. Consistent with this, awareness of the health harms of sugar was generally high among online-surveyed parents, but specific health-risk messages (e.g., infertility, gout, arthritis) were the least persuasive, suggesting that knowledge of harm does not necessarily translate into perceived personal relevance [38].

Several studies demonstrated that food and beverage labelling actively shapes, and frequently distorts, parental knowledge—complementing the perception-level labelling effects reported in Section 3.3. Parents held widespread misperceptions about added sugar content, non-nutritive sweeteners, and juice content in popular children’s drinks [7], and experimental evidence showed that both harmful and corrective labelling could shift this knowledge in either direction: ‘natural’ or ‘no added sweetener’ claims led parents to wrongly believe fruit drinks contained no added sugar [44], whereas warning labels corrected these same misperceptions and improved parents’ understanding of sugar-sweetened beverage options [28].

Educational interventions produced measurable, if sometimes modest, gains in knowledge. A brief educational poster displayed in a paediatric clinic waiting room significantly improved caregiver knowledge of beverage sugar content between the pre- and post-visit surveys [40], and a national media campaign was associated with a short-term reduction in confusion between ‘good’ and ‘bad’ sugar sources, although, as discussed further in Section 3.6, this improvement was not sustained at 12 months [33].

Finally, a subset of studies examined whether higher parental knowledge translated into lower child sugar intake. This evidence was mixed and is examined in depth, alongside the broader relationship between parental cognition and children’s intake, in Section 3.5. Taken together, the findings in this section indicate that parental knowledge gaps are real and modifiable through simple interventions, but that whether improved knowledge actually reduces children’s sugar intake is a separate question, addressed next.

### 3.5. Relationship Between Parental Perceptions/Knowledge and Children’s Sugar Intake

Section 3.3 and Section 3.4 established that parents frequently misjudge the healthfulness of sugar-containing products and often lack accurate factual knowledge about sugar. This section addresses the redefined secondary outcome of this review most directly: to what extent are these cognitive gaps actually related to children’s reported or measured sugar intake? Sixteen of the 35 included studies provided evidence relevant to this question. Rather than measuring a single, uniform ‘discrepancy’, these studies captured the relationship in markedly different ways: as a direct perception-intake gap, as an attitude-behaviour or intention-behaviour gap, as a mediated pathway, or as a direct concordance between parental and child consumption.

The most direct evidence came from studies comparing parental perception against independently measured or objectively verified intake. Parents underestimated their child’s actual sugar-sweetened beverage and food intake relative to independently observed dietary data [23], and, in a separate cohort, parents’ underestimation of their children’s sugar content was associated with more than double the odds of childhood overweight (odds ratio = 2.01), one of the most direct quantitative links in this literature between parental misperception and a child health outcome [26]. These findings extend the perception-level discrepancies already described in Section 3.3 [24,34,49] to objectively verified intake and weight outcomes.

A second, distinct pattern emerged from studies examining attitudes and behavioural intentions rather than perception per se: even clearly held parental attitudes were not reliably associated with actual provision or intake. More negative parental attitudes toward sugar-sweetened beverages were associated with lower infant consumption, yet the large majority of infants in the sample still consumed sugar-sweetened beverages regardless of their parents’ attitude [30]. Using the Theory of Planned Behaviour, one study found that mothers’ explicit intention to reduce added sugar was not associated with their actual sugar-adding behaviour, whereas perceived behavioural control was [48]. Consistent with this attitude-behaviour gap, behavioural change in a Danish cohort was mediated by shifts in parental norms and attitudes rather than by factual knowledge gains, indicating that motivational and normative factors, not cognitive ones, were statistically linked to the observed reductions in intake [51].

The mechanisms linking parental cognition to child intake were examined explicitly in a study using a comprehensive behavioural model incorporating attitudes, subjective norms, and parental modelling, which explained meaningful variance in child intake, although the specific association patterns varied substantially by ethnic group, as discussed further in Section 3.6 [25]. In this model, parental cognition was indirectly associated with child intake through structural and normative factors; the observational design does not establish a direct causal relationship.

A recurring pattern across this domain was that parental behaviour, rather than parental cognition, was frequently more consistently associated with children’s intake, predominantly in cross-sectional and observational designs. Parental sugar-sweetened beverage consumption, not parental or adolescent knowledge of associated health risks, was associated with adolescent sugar-sweetened beverage intake in a large US sample [27]. This behavioural-modelling pattern was replicated in an independent Chinese cohort, where adolescents’ free sugar intake was significantly associated with their parents’ own free sugar intake [47]. A related dyadic study found substantial disagreement between parents and children on the legitimacy of household sugar rules, with 99% of parents but only 26% of children agreeing that such rules were legitimate; notably, structure-based parenting practices were associated with lower measured child intake than coercive control, regardless of this underlying disagreement [37].

Finally, the knowledge-specific studies introduced in Section 3.4—including a longitudinal panel alongside cross-sectional and gold-standard dietary-recall designs—provided methodologically varied, but still inconsistent, evidence on this relationship. In the only two-wave longitudinal panel study in this review, higher parental dietary knowledge was associated with a measurable reduction in children’s sugar-sweetened beverage consumption over time, after accounting for individual fixed effects [52]; a comparable, cross-sectional association was observed in a smaller paediatric clinic sample [29]. However, in a study using the gold-standard method of repeated 24 h dietary recalls, it was children’s own knowledge of sugar recommendations, not their parents’, that was independently associated with lower measured intake, with no correlation between parent and child knowledge scores [35].

Taken as a whole, this body of evidence does not support a simple model in which parental misperception or knowledge gaps directly and uniformly explain children’s excess sugar intake. Instead, the relationship between parental cognition and child intake is heterogeneous: it appears mediated by structural factors such as home availability in the single study that formally tested mediation [25], moderated by cultural and ethnic context, and, in several of the included cross-sectional and observational studies, more consistently associated with parental behavioural modelling than with parental cognition itself. This nuanced picture has direct implications for intervention design, discussed further in Section 4, and connects to the environmental and sociocultural barriers examined next in Section 3.6.

### 3.6. Barriers and Facilitators

If parental cognition alone does not fully explain children’s sugar intake (Section 3.5), the studies in this section point to where the remaining explanatory weight lies: the family, cultural, and commercial environment surrounding sugar provision. Nine of the 35 included studies addressed barriers and facilitators shaping parental provision of sugar to children, spanning qualitative, cross-sectional, and experimental designs. Across this literature, sugar provision was consistently described not as an isolated parental decision but as embedded within family routines, cultural norms, and the broader food environment.

Family celebrations, treats, and inherited habits emerged as the most frequently cited facilitators of sugar provision. Portuguese and Danish parents described sugar as integral to social occasions and reward-based routines, adopting reduction strategies only when compatible with daily family life [9,42]. A similar pattern, with a distinct cultural framing, was reported among Lebanese caregivers, for whom adding sugar to children’s beverages was an inherited, largely unquestioned habit transmitted across generations and reinforced by quasi-medical beliefs about its benefits—for instance, that sugar was “nourishing” or “calming” for infants [50].

Structural and environmental factors also acted as facilitators or barriers depending on context. Frequent restaurant meals and lower caregiver education were associated with higher sugar-sweetened beverage consumption, suggesting that the food environment outside the home compounds parental attitudes [53]. Marketing and product labelling repeatedly emerged as a barrier to informed decision-making: parents reported confusion between product categories (e.g., toddler milk versus infant formula, or fruit drinks versus 100% juice), which they attributed to industry marketing practices [43]. Encouragingly, a randomised trial testing counter-marketing videos addressing these same misperceptions reduced caregivers’ intention to serve sugar-sweetened toddler milks and fruit drinks, with a larger effect observed among Black caregivers for fruit drink outcomes, suggesting that targeted corrective messaging can meaningfully shift intended provision behaviour [45].

At the policy level, parents in Singapore expressed greater support for restricting the availability of sugar-sweetened beverages in specific settings such as schools than for broader fiscal measures such as taxation [39]. A UK national campaign combining media messaging with practical strategies achieved a short-term reduction in children’s sugar intake that was not sustained at 12 months; qualitative interviews conducted alongside the campaign identified “treat culture”, social pressure, and misleading marketing as the principal barriers to sustained change [33].

Finally, the relative weight of these barriers and facilitators varied by sociocultural context. Among Dutch families, a comprehensive model incorporating parental attitudes, subjective norms, and behavioural modelling (introduced in Section 3.5) explained substantially more variance in children’s sugar-sweetened beverage intake among Dutch-origin families than among Moroccan- or Turkish-origin families, for whom different predictors were dominant [25]. Taken together, these findings indicate that reducing children’s sugar exposure is unlikely to be achieved through knowledge-focused interventions alone: family routines, cultural inheritance, marketing exposure, and the wider food environment were each associated with parental provision behaviour, suggesting that interventions may need to address several of these factors simultaneously.

### 3.7. Implications for Oral Health and Caries Prevention

None of the 35 studies included in this review directly assessed dental caries or another oral health outcome; all evidence relevant to caries prevention is therefore indirect, derived from the relationship between parental cognition, parental behaviour, and children’s dietary sugar exposure documented in Section 3.3, Section 3.4, Section 3.5 and Section 3.6. This absence of direct outcome data reflects a deliberate finding of this review—that parental-cognition research on children’s sugar consumption has, to date, rarely been conducted within a dental or cariology framework—rather than an oversight in study selection.

Read against two well-established premises from the cariology literature (see Introduction)—that frequency and amount of fermentable sugar exposure, particularly from sugar-sweetened beverages, are principal modifiable determinants of dental caries risk in children, and that the pattern of exposure, rather than total sugar intake alone, materially affects cariogenic potential—three findings from this review carry direct, if indirect, implications for caries prevention.

First, the consistent parental underestimation of children’s sugar intake documented in Section 3.3 and Section 3.5 (e.g., [23,26]) suggests that many parents may be unaware of the frequency with which their children are exposed to cariogenic substrates, undermining a precondition for effective caries-preventive counselling: parents cannot be expected to modify an exposure they do not accurately perceive. Second, the finding that parental behavioural modelling, rather than parental knowledge or attitude, was more consistently associated with children’s actual intake across several of the included cross-sectional studies in this review [27,37,47] implies that caries-prevention messaging directed solely at increasing parental knowledge is unlikely to be sufficient; interventions that address parental modelling and the home food environment directly (e.g., differences in home availability and family norms across cultural contexts, as identified by [25]) warrant evaluation for their potential to reduce the frequency of cariogenic exposure. Third, the barriers and facilitators identified in Section 3.6, including celebratory and cultural sugar-provision norms and the confounding influence of product marketing and labelling, indicate that caries-preventive interventions embedded solely within dental or paediatric settings may be working against powerful social and commercial determinants that operate largely outside clinical contact.

At the same time, this review’s findings support a cautious, rather than alarmist, framing of parental misperception as a caries risk factor. Several studies (Section 3.5) demonstrated that even accurate parental knowledge or negative attitudes did not consistently translate into lower actual intake, underscoring that parental cognition is only one of several determinants of a child’s cariogenic sugar exposure, alongside household availability, family routines, sociocultural norms, and commercial marketing. Dental caries prevention strategies that rely exclusively on correcting parental misperceptions are therefore unlikely, on the basis of this evidence, to achieve durable reductions in children’s sugar exposure unless they are paired with structural and environmental interventions.

In summary, while no study in this review measured caries directly, the consistent evidence of parental underestimation of sugar exposure, the limited and inconsistent translation of parental knowledge into reduced intake, and the identification of structural and sociocultural barriers to reduction together provide an evidence base for reframing caries-preventive counselling: from a primarily knowledge-transmission model toward one that also addresses behavioural modelling, home food environment, and the sociocultural and commercial context in which parental sugar-provision decisions are made.

## 4. Discussion

### 4.1. Summary of Main Findings

This systematic review synthesised evidence from 35 studies conducted across ten countries between 2016 and 2025. Two broad conclusions emerge. First, parental misperception and knowledge gaps regarding children’s sugar consumption are real, widespread, and consistent across qualitative and quantitative designs (Section 3.3 and Section 3.4): parents systematically overestimate the healthfulness of sugar-containing products, are frequently misled by label claims and marketing, and often lack accurate factual knowledge of sugar content or dietary guidelines. Second, this cognitive gap does not, by itself, adequately explain children’s sugar exposure (Section 3.5): in several of the included cross-sectional and observational studies in this review, parental behavioural modelling—what parents themselves eat and drink—and structural or environmental factors were more consistently associated with children’s intake than parental perception, attitude, or knowledge; the review’s small number of experimental and longitudinal studies precludes firm causal or comparative claims (Section 4). Parental cognition is therefore relevant but insufficient on its own to explain children’s sugar exposure; behavioural modelling and the wider food environment appear to be similarly relevant correlates.

No study included in this review measured dental caries or another oral health outcome directly (Section 3.7); consequently, all clinical implications discussed below remain inferential, grounded in the well-established relationship between fermentable sugar exposure and cariogenic biofilm dysbiosis rather than in direct empirical evidence linking parental cognition to caries incidence. This absence of direct outcome data is a feature of the evidence base as a whole, not a limitation specific to this review’s search strategy, and is addressed explicitly rather than treated as an incidental gap. The evidentiary base is also heterogeneous in design and strength: the large majority of studies are cross-sectional or qualitative and therefore support only associational inferences about parental cognition and sugar exposure, whereas a smaller number of intervention or quasi-experimental studies (corrective warning labels, educational posters, counter-marketing videos, and media campaigns [28,33,40,44,45]) provide experimental or quasi-experimental, though still indirect, evidence that specific messaging or labelling can change parental understanding and, in some cases, short-term provision behaviour. These two evidence types are not interchangeable: the observational associations documented elsewhere in this review should not be read as evidence that modifying parental cognition alone would causally reduce children’s sugar intake, a claim only the intervention studies can partially and provisionally support.

### 4.2. Beyond the Perception Gap: Parental Cognition, Behaviour, and Children’s Sugar Exposure

The perception-reality gap that anchored the original version of this review remains well supported: across nine studies, parents and caregivers consistently misjudged the healthfulness of sugar-containing foods and beverages (Section 3.3), and across thirteen further studies, factual knowledge of sugar content and dietary guidelines was frequently limited (Section 3.4). When perception and objectively measured or independently verified intake were compared directly, the gap was substantial and, in at least one study, associated with more than double the odds of childhood overweight [26].

However, the expanded evidence base in this review complicates a purely cognitive account of children’s sugar exposure. Sixteen studies examined the relationship between parental perception or knowledge and children’s actual intake, and this relationship proved markedly heterogeneous (Section 3.5). Clearly held parental attitudes did not reliably translate into lower provision or intake [30], explicit behavioural intentions did not predict actual sugar-adding behaviour once perceived behavioural control was accounted for [48], and in mediation analysis conducted in one study, parental attitude was indirectly associated with child intake through structural factors such as home availability in the study’s mediation model [25]. Most notably, in several of the largest cross-sectional studies in this review, it was parental behaviour itself—not parental knowledge, attitude, or perception—that was most consistently associated with children’s actual sugar intake [27,47,49]; because these designs are predominantly cross-sectional, these associations should not be interpreted as established causal effects.

This pattern converges with, and substantially extends, Bestle et al.’s (2025) [51] demonstration that behavioural change was mediated by shifts in parental norms and attitudes rather than by factual knowledge gains. The present review corroborates this conclusion with independent evidence from multiple countries and study designs, and adds a complementary mechanism—direct behavioural modelling—that reinforces the same underlying conclusion: models of parental dietary gatekeeping that treat accurate parental perception as the primary lever for reducing children’s sugar exposure are incomplete. Several observational studies were consistent with a behavioural-modelling pathway, but their designs do not establish that this pathway operates independently or causally.

### 4.3. Nutritional Misperceptions, Labelling, and Cariogenic Risk

The mechanisms associated with parental misperception in this review may be relevant to caries prevention through their potential influence on sugar exposure. Descriptors such as ‘natural’, ‘made with fruit’, or ‘no added sweetener’ were associated with a ‘health halo’ and with parents judging sugar-sweetened beverages as healthier than they were, independent of actual sugar content [36,44,46]; label-driven perception, rather than actual composition, was also associated with provision decisions in both directions [41]. For dental caries, the distinction between ‘natural’ and ‘added’ sugar that parents frequently draw on is biologically irrelevant: the cariogenic potential of free sugars is equivalent regardless of source once in contact with a susceptible tooth surface in the presence of cariogenic biofilm. Widespread parental misperceptions about added sugar, non-nutritive sweeteners, and juice content in popular children’s drinks [7] indicate a nutritional literacy deficit that dental professionals are well positioned to address during routine preventive consultations, and experimental evidence in this review confirms that this deficit is directly modifiable: corrective warning labels measurably improved parental understanding of sugar-sweetened beverage options [28], and counter-marketing videos reduced caregivers’ intention to serve sugar-sweetened toddler milks and fruit drinks [45].

The biological pathway linking sustained parental misperception to dental caries operates through cumulative exposure rather than through any single decision: when caregivers underestimate or misclassify cariogenic products, children’s teeth experience more frequent and prolonged exposure to fermentable carbohydrates, driving a shift in the oral biofilm toward acidogenic, aciduric species such as Streptococcus mutans and progressive enamel demineralisation [12,13,56]. The perception–reality gap may therefore be relevant to oral microbial ecology through repeated sugar exposure [57]; however, this pathway is inferential because none of the included studies measured caries or oral microbiological outcomes directly.

### 4.4. Environmental, Cultural, and Commercial Determinants

A further consequence of the expanded evidence base is a substantially richer picture of why parental cognition, even when accurate, often fails to translate into reduced sugar provision. Sugar remains deeply embedded in family celebration and reward routines across culturally distinct settings, from Portuguese and Danish families adopting reduction strategies only when compatible with daily life [9,42] to Lebanese caregivers for whom adding sugar to infant beverages is an inherited, largely unquestioned practice reinforced by quasi-medical beliefs about its benefits [50]. Structural features of the food environment compound these cultural norms: frequent restaurant meals and lower caregiver education were independently associated with higher sugar-sweetened beverage intake [53], and product marketing was repeatedly reported as a source of parental confusion between product categories, independent of any underlying knowledge deficit [43].

These findings have a direct bearing on intervention design and policy advocacy. A UK national media campaign achieved a measurable short-term reduction in children’s sugar intake that was not sustained at 12 months, with qualitative data identifying ‘treat culture’, social pressure, and misleading marketing—not residual lack of knowledge—as the principal barriers to durable change [33]. Consistent with this, parents in this review expressed greater support for restricting the availability of sugar-sweetened beverages in specific settings such as schools than for broader fiscal measures such as sugar taxation [39], a preference dental and public health advocates should take into account when selecting policy levers likely to secure caregiver buy-in. Taken together, the evidence in this review indicates that reducing children’s sugar exposure is unlikely to be achieved through knowledge-focused interventions alone, however well-designed; family routines, cultural inheritance, marketing exposure, and the wider food environment were each associated with parental provision behaviour in the included evidence.

### 4.5. Implications for Dental Practice and Public Health

The findings of this review support a reframing of caries-preventive counselling away from a primarily knowledge-transmission model. Dietary history-taking in clinical practice should capture not only the child’s reported sugar exposure but also the accompanying adult’s own consumption habits, given the recurring observational association with behavioural modelling identified in Section 3.5. Label literacy training should address ‘natural’ and ‘no added sweetener’ claims specifically, since these were the most consistently identified correlates of parental misperception in this review, and brief, low-cost educational interventions of the kind shown here to produce measurable short-term knowledge gains [40] remain worthwhile, provided expectations about their limited durability absent reinforcement of the home food environment are realistic. Because barriers to reduction operate substantially outside the dental setting, collaborative referral pathways with paediatricians, dietitians, and school health services are warranted, alongside advocacy for environmental and school-based restriction policies, which this review’s evidence suggests caregivers are considerably more receptive to than fiscal measures such as taxation.

More broadly, this reframing does not diminish the clinical relevance of nutritional education—the perception and knowledge gaps documented in this review remain real, common, and clinically actionable—but it does argue against treating knowledge transmission as a sufficient intervention. Counselling that engages caregivers’ own dietary behaviour and the home food environment, alongside factual education, is better aligned with the totality of evidence synthesised here than counselling that addresses parental knowledge in isolation.

### 4.6. Strengths and Limitations

This review has several strengths. The evidence base draws on 35 studies across ten countries, identified through a systematic search of five databases (PubMed, Scopus, PsycINFO, CINAHL, and Web of Science) spanning 2016 to 2026. Parental perceptions, beliefs, attitudes, and knowledge are treated as a single, coherently defined primary outcome, with their relationship to children’s reported or measured sugar intake assessed as a distinct secondary outcome, allowing the full set of included studies to be organised within one consistent outcome hierarchy rather than assessed against a single, narrowly defined comparison. Risk-of-bias appraisal was based on independent full-text review for all included studies, using design-appropriate JBI critical-appraisal checklists and the Cochrane RoB 2 tool.

Several limitations remain. Heterogeneity in designs, constructs, exposures, and outcome measures precluded meta-analysis and limited the synthesis to structured narrative comparison. Dietary and behavioural outcomes were usually self-reported and were vulnerable to recall and social-desirability bias. Consumption measures varied in format and reference period, limiting direct comparability. Multiple studies used bespoke or modified questionnaires without complete psychometric validation; these studies were retained because instrument validity was treated as an appraisal issue rather than an eligibility criterion, but their findings warrant additional caution. The evidence was concentrated in the United States, and restriction to English- and Spanish-language reports limits generalisability. The January 2016 lower limit was selected to provide an approximately decade-long contemporary synthesis beginning after the WHO 2015 sugar-intake guideline and situated within the subsequent food-labelling and beverage-marketing environment; however, it was a pragmatic restriction and may have excluded relevant earlier evidence. Likewise, although pilot searches suggested substantial overlap for “added sugar*” and “soda” with the retained sugar-specific terms, the strategy did not include every possible beverage synonym independently, which may have reduced sensitivity. The protocol was not prospectively registered, and the authors had been exposed to results from an initial PubMed/Scopus search before the framework was clarified and the search expanded. Although the final five-database record set was screened using the clarified criteria, this sequence cannot eliminate the possibility that emerging evidence influenced methodological decisions. Finally, even with complete item-level appraisal, the design-specific instruments cannot be converted validly into a single pooled risk-of-bias distribution. Family norms, parenting practices, household availability, exposure frequency, and cultural and commercial context were associated with sugar exposure in different settings, but the evidence does not permit formal weighting of their relative contribution.

The most important limitation for clinical interpretation is that no included study measured dental caries or any other direct clinical oral-health outcome. Consequently, this review cannot establish a direct relationship between parental cognition or behaviour and caries incidence, severity, or prevention. All caries-related implications remain indirect extrapolations from evidence on children’s dietary sugar exposure and from the established biological relationship between fermentable sugars and caries. This limitation substantially restricts the clinical certainty of the conclusions and should be addressed in future longitudinal and intervention studies incorporating objective dietary measures and clinical caries outcomes.

## 5. Conclusions

Across the included evidence, caregivers’ own dietary behaviour, parental modelling, household availability, family routines, and the wider food environment were recurring correlates of children’s sugar intake. Parental misperceptions and knowledge gaps were also common, but the predominantly observational evidence does not establish that correcting cognition alone would reduce children’s sugar exposure.

Caries-preventive counselling should therefore combine accurate sugar-related information with strategies addressing caregiver behaviour and the home food environment. Because none of the included studies assessed dental caries or another direct clinical oral-health outcome, these preventive implications remain indirect. Future research should prioritise longitudinal and intervention designs that link parental perceptions, caregiver behaviour, and home-environment measures to objective dietary and clinical caries outcomes.

## Figures and Tables

**Table 1:** Characteristics of the 35 included studies, ordered chronologically by publication year and alphabetically by first author within each year.

No.	Study	Country	Design	Sample	Child Age	Parental Construct	Assessment Method	Child Dietary Outcome	Main Finding	Domain(s)
1	Munsell et al. 2016 [8]	USA	Online survey	982 parents	Not reported	Belief	Questionnaire (validation not reported)	No direct dietary outcome	A substantial proportion of parents believed certain sugar-sweetened beverages were healthy.	Perception
2	Van de Gaar et al. 2016 [23]	Netherlands	Cross-sectional	275 dyads	School-aged children	Perception	Mixed methods	SSB intake	Parents underestimated their child’s actual SSB/food intake relative to independently observed data.	Relationship
3	Zytnick, Park and Onufrak 2016 [24]	USA	Cross-sectional	815 dyads	12–17 y	Attitude	Questionnaire (validation not reported)	SSB intake	Parental beliefs that sports drinks were ‘healthy’ or ‘necessary for hydration’ were more strongly associated with adolescent consumption than adolescents’ own attitudes were.	Perception; Relationship
4	van de Gaar et al. 2017 [25]	Netherlands (Rotterdam)	Cross-sectional (TPB)	644 dyads	6–13 y	Multiple	Validated questionnaire	SSB intake	Parental attitudes, norms, and modelling were associated with SSB intake, with markedly different association patterns across ethnic groups.	Relationship; Barriers/Facilitators
5	Zahid, Davey and Reicks 2017 [6]	USA (Minnesota)	Cross-sectional	194 dyads	9–12 y	Knowledge	Validated questionnaire	SSB intake	Home beverage availability was associated with SSB intake; parental sugar knowledge was linked to more dairy consumption but not less SSB intake.	Knowledge
6	Dallacker et al. 2018 [26]	Germany	Cross-sectional	305 dyads	Not reported	Knowledge	Questionnaire (validation not reported)	Total sugar intake	Parents substantially underestimated the sugar content of their children’s diet, associated with higher child overweight risk.	Relationship
7	Lundeen et al. 2018 [27]	USA	Cross-sectional	990 dyads	12–17 y	Knowledge	Questionnaire (validation not reported)	SSB intake	Parental SSB consumption, not parental or adolescent knowledge of health risks, was associated with adolescent SSB intake.	Knowledge; Relationship
8	Moran & Roberto 2018 [28]	USA	Randomised experiment	2381 parents	Not reported	Perception	Experimental task	No direct dietary outcome	Warning labels corrected parental misperceptions about the healthfulness of sugar-sweetened beverage options.	Knowledge
9	Sangiovanni et al. 2018 [29]	USA	Cross-sectional	52	2–12 y	Knowledge	Questionnaire (validation not reported)	SSB intake	Only 14% of parents correctly identified AAP juice guidelines; higher knowledge was independently associated with lower child juice/SSB intake.	Knowledge; Relationship
10	Woo Baidal et al. 2018 [30]	USA	Cross-sectional	394 WIC families	0–24 months	Attitude	Validated questionnaire	SSB intake	More negative parental attitudes toward SSBs were associated with lower infant SSB consumption, though most infants still consumed SSBs regardless.	Relationship
11	Kim et al. 2019 [31]	USA	Cross-sectional	55 caregivers	Not reported	Knowledge	Questionnaire (validation not reported)	No direct dietary outcome	Caregivers reported limited knowledge and inconsistent perceived control over their child’s sugar/SSB intake.	Knowledge
12	Smith et al. 2019 [32]	USA	Interview-based survey	100 parents	Not reported	Belief	Semi-structured interview	No direct dietary outcome	Parents preferred sugar over non-nutritive sweeteners for their children, a preference they attributed to fear of adverse effects from sweeteners.	Perception
13	Bradley et al. 2020 [33]	UK	Pre-post (government campaign)	Not reported	5–11 y	Belief	Mixed methods	Total sugar intake	A national campaign produced a short-term reduction in children’s sugar intake, not sustained at 12 months; parents cited treat culture and marketing as barriers.	Knowledge; Barriers/Facilitators
14	Eliason et al. 2020 [34]	USA	Cross-sectional	2229 households	3–18 y	Perception	Questionnaire (validation not reported)	Total sugar intake	Parents overestimated the healthfulness of their children’s diets relative to actual sugar intake.	Perception; Relationship
15	Justiz et al. 2020 [35]	USA (Texas, TX Sprouts)	Cross-sectional (RCT baseline)	685 dyads	7–12 y	Knowledge	Mixed methods	Total sugar intake	Children’s own knowledge of sugar recommendations, not their parents’, was associated with lower sugar intake.	Knowledge; Relationship
16	Miller et al. 2020 [36]	Australia	Qualitative (focus groups)	59 (27 young adults + 30 parents)	0–12 y	Perception	Focus group	No direct dietary outcome	Daily sugary drink consumption was normalised by parents, who showed limited awareness of actual sugar content.	Perception
17	Thomson et al. 2020 [37]	USA	Cross-sectional (FLASHE)	1657 dyads	Not reported	Belief	Validated questionnaire	Total sugar intake	Parents and children frequently disagreed on the legitimacy of household sugar rules; structure-based practices were linked to lower child intake than coercive control.	Relationship
18	Carl et al. 2021 [38]	USA	Cross-sectional (online)	1058	2–12 y	Knowledge	Questionnaire (validation not reported)	No direct dietary outcome	Awareness of sugar-related health harms was high, but messages about infertility, gout and arthritis were least persuasive to parents.	Knowledge
19	Chan et al. 2021 [39]	Singapore	Qualitative	20 parents	3–5 y	Multiple	Semi-structured interview	No direct dietary outcome	Parents favoured SSB availability restrictions in specific settings (e.g., schools) over broader taxation policies.	Barriers/Facilitators
20	Harris & Pomeranz 2021 [7]	USA	Experimental + cross-sectional	1603 parents	1–5 y	Multiple	Mixed methods	No direct dietary outcome	Parents held widespread misperceptions about added sugar, non-nutritive sweeteners, and juice content in popular children’s drinks.	Knowledge
21	Lee et al. 2021 [40]	USA	Pre-post (educational poster)	149	0–18 y	Knowledge	Study-specific pre/post questionnaire; validation not reported	No direct dietary outcome	A brief educational poster significantly improved caregiver knowledge of beverage sugar content from pre- to post-visit.	Knowledge
22	Prada et al. 2021 [9]	Portugal	Qualitative	42 parents	6–10 y	Multiple	Semi-structured interview	No direct dietary outcome	Parents identified specific beliefs, practices and barriers shaping their children’s sugar intake, including celebrations and family habits.	Barriers/Facilitators
23	Choi et al. 2022 [41]	USA	Cross-sectional	1671 caregivers	1–5 y	Multiple	Validated questionnaire	Food provision	Perceived healthfulness and specific label claims (e.g., ‘vitamin C’) were associated with more frequent provision of sweetened drinks to young children.	Perception; Knowledge
24	Christensen et al. 2022 [42]	Denmark	Qualitative	24 families	School-aged children	Multiple	Semi-structured interview	No direct dietary outcome	Sugar was embedded in family routines and celebrations; parents used context-dependent strategies to reduce intake.	Barriers/Facilitators
25	Fleming-Milici, Phaneuf, and Harris 2022 [43]	USA	Qualitative (9 focus groups)	50 parents	9–36 months	Perception	Focus group	No direct dietary outcome	Parents reported that marketing claims contributed to confusion about product categories and ingredients and to beliefs that sweetened drinks were healthy or necessary.	Barriers/Facilitators
26	Hall et al. 2022 [44]	USA	Randomised trial (virtual store)	2219 parents	1–5 y	Belief	Experimental task	Food provision	‘Natural’ and ‘no added sweetener’ claims led parents to wrongly believe fruit drinks contained no added sugar and to select less healthy options.	Knowledge
27	Harris et al. 2022 [45]	USA	RCT	600	9–36 months	Belief	Experimental task	Food provision	Counter-marketing videos reduced caregivers’ intention to serve toddler milks and fruit drinks, with larger effects among Black caregivers for fruit drinks.	Barriers/Facilitators
28	Sylvetsky et al. 2022 [46]	USA	Cross-sectional	Not reported	Not reported	Perception	Questionnaire (validation not reported)	No direct dietary outcome	Certain beverage attributes (e.g., ‘natural’, ‘made with fruit’) generated a false perception of healthfulness among parents.	Perception
29	Zhang et al. 2022 [47]	China (Changsha)	Cross-sectional	1090 adolescents	Not reported	Provision practices	Questionnaire (validation not reported)	Total sugar intake	Adolescents’ free sugar intake was significantly associated with their parents’ own free sugar intake.	Relationship
30	Abdel Rahman et al. 2024 [48]	Lebanon	Cross-sectional (TPB)	184 mothers	3–7 y	Attitude	Validated questionnaire	Total sugar intake	Mothers’ intention to reduce added sugar was not associated with actual behaviour; perceived behavioural control was the only significant predictor in the model.	Relationship
31	Talati et al. 2024 [49]	Australia	Cross-sectional	1611 dyads	School-aged children	Perception	Validated questionnaire	Total sugar intake	Children perceived flavoured milk and juice as healthier than they were; parents’ own consumption, not their perception, was associated with their child’s intake.	Perception; Relationship
32	Abdel Rahman et al. 2025 [50]	Lebanon	Qualitative (26 interviews)	26 (16 mothers, 8 grandmothers, 2 fathers)	9–12 y	Belief	Semi-structured interview	No direct dietary outcome	Adding sugar to children’s beverages was a deeply ingrained social norm that participants associated with quasi-medical beliefs about its benefits and described as passed down across generations.	Barriers/Facilitators
33	Bestle et al. 2025 [51]	Denmark	Mediation analysis	153 dyads	5–7 y	Attitude	Questionnaire with cognitive testing, field testing, CFA, and internal-consistency assessment	Total sugar intake	Reductions in children’s sugar intake were mediated by changes in parental norms and attitudes, not by factual knowledge gains.	Relationship
34	Cui et al. 2025 [52]	China	Longitudinal (2-wave panel)	3962 students	School-aged children	Knowledge	Validated questionnaire	SSB intake	Higher parental dietary knowledge was longitudinally associated with reduced children’s SSB consumption, controlling for individual fixed effects.	Knowledge; Relationship
35	Fuster et al. 2025 [53]	USA (Louisiana)	Cross-sectional	1006 caregivers	2–12 y	Attitude	Questionnaire (validation not reported)	SSB intake	Frequent SSB consumption was associated with more restaurant meals, lower caregiver education, and pro-SSB attitudes.	Relationship; Barriers/Facilitators

AAP, American Academy of Pediatrics; RCT, randomised controlled trial; SSB, sugar-sweetened beverage; TPB, Theory of Planned Behaviour; WIC, Special Supplemental Nutrition Program for Women, Infants, and Children. “Multiple” indicates that more than one parental construct (perception, belief, attitude, knowledge, and/or nutritional literacy) was assessed within the same study. “No direct dietary outcome” indicates that the study assessed parental cognition or provision practices without reporting a separate measured or reported child-intake outcome.

**Table 2:** Tool-specific risk-of-bias appraisal for the 35 included studies, ordered as in Table 1.

No.	Study	Tool	Reporting Approach/Official RoB 2 Judgement
1	Munsell et al. 2016 [8]	JBI analytical cross-sectional	Item-level JBI appraisal; no global category assigned
2	Van de Gaar et al. 2016 [23]	JBI analytical cross-sectional	Item-level JBI appraisal; no global category assigned
3	Zytnick, Park, and Onufrak 2016 [24]	JBI analytical cross-sectional	Item-level JBI appraisal; no global category assigned
4	van de Gaar et al. 2017 [25]	JBI analytical cross-sectional	Item-level JBI appraisal; no global category assigned
5	Zahid, Davey, and Reicks 2017 [6]	JBI analytical cross-sectional	Item-level JBI appraisal; no global category assigned
6	Dallacker et al. 2018 [26]	JBI analytical cross-sectional	Item-level JBI appraisal; no global category assigned
7	Lundeen et al. 2018 [27]	JBI analytical cross-sectional	Item-level JBI appraisal; no global category assigned
8	Moran and Roberto 2018 [28]	Cochrane RoB 2	Some concerns
9	Sangiovanni et al. 2018 [29]	JBI analytical cross-sectional	Item-level JBI appraisal; no global category assigned
10	Woo Baidal et al. 2018 [30]	JBI analytical cross-sectional	Item-level JBI appraisal; no global category assigned
11	Kim et al. 2019 [31]	JBI analytical cross-sectional	Item-level JBI appraisal; no global category assigned
12	Smith et al. 2019 [32]	JBI analytical cross-sectional	Item-level JBI appraisal; no global category assigned
13	Bradley et al. 2020 [33]	JBI quasi-experimental	Item-level JBI appraisal; no global category assigned
14	Eliason et al. 2020 [34]	JBI analytical cross-sectional	Item-level JBI appraisal; no global category assigned
15	Justiz et al. 2020 [35]	JBI analytical cross-sectional	Item-level JBI appraisal; no global category assigned
16	Miller et al. 2020 [36]	JBI qualitative	Item-level JBI appraisal; no global category assigned
17	Thomson et al. 2020 [37]	JBI analytical cross-sectional	Item-level JBI appraisal; no global category assigned
18	Carl et al. 2021 [38]	JBI analytical cross-sectional	Item-level JBI appraisal; no global category assigned
19	Chan et al. 2021 [39]	JBI qualitative	Item-level JBI appraisal; no global category assigned
20	Harris and Pomeranz 2021 [7]	Cochrane RoB 2	Some concerns
21	Lee et al. 2021 [40]	JBI quasi-experimental	Item-level JBI appraisal; no global category assigned
22	Prada et al. 2021 [9]	JBI qualitative	Item-level JBI appraisal; no global category assigned
23	Choi et al. 2022 [41]	JBI analytical cross-sectional	Item-level JBI appraisal; no global category assigned
24	Christensen et al. 2022 [42]	JBI qualitative	Item-level JBI appraisal; no global category assigned
25	Fleming-Milici, Phaneuf, and Harris 2022 [43]	JBI qualitative	Item-level JBI appraisal; no global category assigned
26	Hall et al. 2022 [44]	Cochrane RoB 2	Low risk
27	Harris et al. 2022 [45]	Cochrane RoB 2	High risk
28	Sylvetsky et al. 2022 [46]	JBI analytical cross-sectional	Item-level JBI appraisal; no global category assigned
29	Zhang et al. 2022 [47]	JBI analytical cross-sectional	Item-level JBI appraisal; no global category assigned
30	Abdel Rahman et al. 2024 [48]	JBI analytical cross-sectional	Item-level JBI appraisal; no global category assigned
31	Talati et al. 2024 [49]	JBI analytical cross-sectional	Item-level JBI appraisal; no global category assigned
32	Abdel Rahman et al. 2025 [50]	JBI qualitative	Item-level JBI appraisal; no global category assigned
33	Bestle et al. 2025 [51]	JBI cohort	Item-level JBI appraisal; no global category assigned
34	Cui et al. 2025 [52]	JBI cohort	Item-level JBI appraisal; no global category assigned
35	Fuster et al. 2025 [53]	JBI analytical cross-sectional	Item-level JBI appraisal; no global category assigned

JBI, Joanna Briggs Institute; RoB 2, Cochrane Risk of Bias 2 tool. RoB 2 retains its official overall judgements. JBI item-level findings are reported in Table S3 and are not converted into review-defined global categories. The 11-item JBI cohort checklist was applied without modification to the two longitudinal studies.

## Data Availability

All data extracted for this review that support the reported synthesis are presented in the manuscript and Supplementary Materials. The verbatim search strategies, amendment history, and available item-level appraisal matrix are provided in Tables S1–S3. No analytic code was used.

## Supplementary Materials

The following supporting information can be downloaded at: https://www.mdpi.com/article/10.3390/children13091236/s1. Table S1: Complete database-specific search strategies, search fields, limits, search dates, retrieval yields, and rationale for search-term selection; Table S2: Amendment history documenting changes to the review methodology and eligibility framework; Table S3: Study-level risk-of-bias appraisal, including item- or domain-level judgements and study-specific justifications.

## Abbreviations

The following abbreviations are used in this manuscript: SSB: Sugar-Sweetened Beverage; JBI: Joanna Briggs Institute; RoB 2: Cochrane Risk of Bias 2 tool for randomised trials; PICo: Population, Interest, Context; PRISMA: Preferred Reporting Items for Systematic Reviews and Meta-Analyses; PROSPERO: International Prospective Register of Systematic Reviews; CINAHL: Cumulative Index to Nursing and Allied Health Literature; ECC: Early Childhood Caries; WHO: World Health Organization.
