# Supplementary material for: Parental Cognition Regarding Children’s Sugar Consumption and Its Implications for Dental Caries Prevention: A Systematic Review
Source: Children (Basel). 2026 Sep 12;13(9):1236. doi: 10.3390/children13091236 (PMC13605704; doi:10.3390/children13091236)
Supplement: Supplementary file 1 [file children-13-01236-s001.zip › children-4531282-supplementary.pdf]

Supplementary Materials

Parental Cognition Regarding Children's Sugar Consumption and Its Impli.cations for Dental Caries Prevention: A Systematic Review

Table S1. Complete search strategy for each database.

Database	Search strategy, filters, and search date
PubMed	((“sugar intake”[Title/Abstract] OR “sugar consumption”[Title/Abstract] OR “free sugar*”[Title/Abstract] OR “sugar-sweetened beverage*”[Title/Abstract] OR “sugary drink*”[Title/Abstract] OR “sweet snack*”[Title/Abstract]) AND (parent*[Title/Abstract] OR caregiver*[Title/Abstract]) AND (perception*[Title/Abstract] OR belief*[Title/Abstract] OR attitude*[Title/Abstract] OR practice*[Title/Abstract] OR knowledge[Title/Abstract] OR norm*[Title/Abstract] OR misperception*[Title/Abstract]) AND (child*[Title/Abstract] OR school-aged[Title/Abstract] OR “young children”[Title/Abstract] OR preschool*[Title/Abstract]))<br>Filters: Date of publication 01/01/2016–04/24/2026. No language filter applied at search stage (language screened at eligibility).<br>Records retrieved: n = 320.<br>Search date: 24 April 2026.
Scopus	TITLE-ABS-KEY ((“sugar intake” OR “sugar consumption” OR “free sugar*” OR “sugar-sweetened beverage*” OR “sugary drink*” OR “sweet snack*”) AND (parent* OR caregiver*) AND (perception* OR belief* OR attitude* OR practice* OR knowledge OR norm* OR misperception*) AND (child* OR school-aged OR “young children” OR preschool*)) AND PUBYEAR > 2015 AND (LIMIT-TO (LANGUAGE , “English”) OR LIMIT-TO (LANGUAGE , “Spanish”))<br>Filters: Publication year 2016–2026; document type: Article; language: English or Spanish.<br>Records retrieved: n = 48.<br>Search date: 24 April 2026.
PsycINFO (EBSCOhost)	(TX (“sugar intake” OR “sugar consumption” OR “free sugar#” OR “sugar-sweetened beverage#” OR “sugary drink#” OR “sweet snack#”) OR DE “Sugars”) AND (TX (parent# OR caregiver#) OR DE “Parents” OR DE “Caregivers”) AND (TX (perception# OR belief# OR attitude# OR practice# OR knowledge OR norm# OR misperception#) OR DE “Parental Attitudes” OR DE “Parent Child Relations”) AND (TX (child# OR “school-aged” OR “young children” OR preschool#) OR DE “Childhood Development”)<br>Filters: Published Date 20160101–20260424. No language filter applied at search stage.<br>Records retrieved: n = 64.<br>Search date: 24 April 2026.
CINAHL (EBSCOhost)	(TX (“sugar intake” OR “sugar consumption” OR “free sugar#” OR “sugar-sweetened beverage#” OR “sugary drink#” OR “sweet snack#”) OR MH “Dietary Sugars”) AND (TX (parent# OR caregiver#) OR MH “Parents” OR MH “Caregivers”) AND (TX (perception# OR belief# OR attitude# OR practice# OR knowledge OR norm# OR misperception#) OR MH “Health Knowledge, Attitudes, Practice”) AND (TX (child# OR “school-aged” OR “young children” OR preschool#) OR MH “Child”)<br>Filters: Published Date 20160101–20260424. No language filter applied at search stage.<br>Records retrieved: n = 136.<br>Search date: 24 April 2026.
Web of Science Core Collection	TS=((“sugar intake” OR “sugar consumption” OR “free sugar*” OR “sugar-sweetened beverage*” OR “sugary drink*” OR “sweet snack*”) AND (parent* OR caregiver*) AND (perception* OR belief* OR attitude* OR practice* OR knowledge OR norm* OR misperception*) AND (child* OR “school-aged” OR “young children” OR preschool*)

Database	Search strategy, filters, and search date
	)<br>Filters: Timespan 2016-01-01 to 2026-04-24; Database: Web of Science Core Collection. No language filter applied at search stage.<br>Records retrieved: n = 793.<br>Search date: 24 April 2026.

Note: The January 2016 lower limit defined an approximately decade-long contemporary evidence window beginning immediately after publication of the WHO's 2015 sugar-intake guideline. It was selected to prioritise evidence generated within the subsequent food-labelling, beverage-marketing, and public-health context, not because earlier studies were considered irrelevant; exclusion of pre-2016 evidence remains a limitation. During strategy development, "added sugar*" and "soda" were considered as independent terms, but pilot searches indicated substantial overlap with records already captured by the broader truncated terms "sugar-sweetened beverage*" and "sugary drink*". Broader beverage terms without an explicit sugar qualifier, such as "soft drink*", were not added independently in order to preserve the review's focus on sugar-specific parental cognition rather than general beverage intake. Omission of additional independent synonyms may nevertheless have reduced sensitivity. Retrieval volumes across databases are not directly comparable because database coverage, indexing practices, search fields, and the availability or application of filters differed across platforms; the larger Web of Science yield should therefore not be attributed solely to broader indexing. All five databases were searched on 24 April 2026; records were combined into a single reference-management library and deduplicated together, as described in Section 2.3 of the manuscript.

Table S2. Amendment table: summary of methodological changes across revision rounds.

Revision round	Change made	Rationale
Original submission → First revision	Search expanded from two databases (PubMed, Scopus) to five databases (PubMed, Scopus, PsycINFO, CINAHL, Web of Science Core Collection); research question and eligibility criteria restructured around a PICo framework; risk-of-bias assessment re-run with independent, duplicate review at every stage for all included studies.	In response to first-round reviewer comments requesting broader database coverage and a more rigorous, reproducible methodology.
First revision → Second revision	Table 1 reordered chronologically by publication year and alphabetically by first author within each year; unnecessary columns removed from Table 2; Figure 2 redesigned as a standard traffic-light risk-of-bias plot with an accompanying summary bar chart.	In response to reviewer comments on table length/ordering, redundant columns, and non-standard figure format.
Second revision → Current (Major Revision) round	The manuscript now reports the initial PubMed/Scopus search of 2 April 2026 and the expanded five-database search of 24 April 2026. The final counts derive from the unified record set generated on 24 April.	To document the actual sequence transparently and acknowledge that protocol clarification occurred after exposure to part of the evidence base.
Current (Major Revision) round	Two studies (Qiu et al., 2021; Killion et al., 2023) removed from the 37 previously included studies following full re-verification against the finalised eligibility criteria: one study's sample population extended beyond the review's 0–18-year age range without an extractable subgroup, and one study constituted a secondary thematic synthesis of previously published qualitative research rather than primary caregiver data. All tables, figures, reference numbering, and summary counts were updated accordingly (35 studies now included).	In direct response to the reviewer's identification of these two eligibility inconsistencies in Table 1.
Current (Major Revision) round	Introduction, Objectives, Eligibility Criteria, Results, Discussion, and Limitations sections revised to: soften unsupported "direct caries" language in favour of sugar-exposure framing; explicitly distinguish the primary from the secondary objective; explicitly distinguish parental mental models from children's actual sugar intake in the eligibility criteria; signpost the separation between cognition-focused and behavioural/environmental findings in Results; add an explicit hierarchy-of-evidence discussion distinguishing observational from intervention/quasi-experimental studies; and expand the Limitations section to address dietary-measure comparability, questionnaire validation status, and the multi-factorial, non-weighted nature of the determinants identified.	In direct response to Reviewer 1's structural and content comments.
Current (Major Revision) round	Complete search strategies for all five databases (Table S1) and a completed PRISMA 2020 checklist provided as supplementary material.	In direct response to Reviewer 2's request for full reproducible search documentation and a completed PRISMA 2020 checklist.
Current technical revision	The previous traffic-light risk-of-bias plot and accompanying summary	To align the presentation with the design-specific appraisal methods and

Revision round	Change made	Rationale
	bar chart were removed. Risk-of-bias findings are now reported item by item using the relevant design-specific JBI checklist or Cochrane RoB 2. JBI assessments are not converted into review-defined global categories, and no pooled summary is presented across non-equivalent tools.	avoid unsupported aggregation across JBI and RoB 2 instruments, as requested by Reviewer 2.
Current editorial minor revision	Section 4.6 now identifies the absence of direct clinical dental-caries outcomes as the principal limitation for clinical interpretation. Section 2.2 and Table S1 provide an expanded rationale for the 2016 lower limit and for retaining sugar-specific search terminology without retrospectively altering the completed search. The Conclusions now foreground caregiver behaviour, parental modelling, household availability, family routines, and the home food environment.	In response to the editor's requests to strengthen the clinical limitation, justify the time window and term selection rigorously, and refocus the Conclusions on behavioural and home-environment evidence.

Table S3. Item-level risk-of-bias appraisal for the 35 included studies.

Methodological note: Every judgement below was re-checked against the complete study report. JBI responses are Y (yes), N (no), U (unclear), or NA (not applicable). JBI instruments do not generate an official overall risk-of-bias category; none has been imposed. RoB 2 uses L (low risk), SC (some concerns), and H (high risk), with its official overall judgement. Bestle et al. and Cui et al. were evaluated as longitudinal studies with the 11-item JBI cohort checklist; Q11 is reported in the final column. Study-specific reasons are supplied so that every judgement is reproducible.

Table S3A. JBI analytical cross-sectional checklist.

No.	Study	Q1	Q2	Q3	Q4	Q5	Q6	Q7	Q8
1	Munsell et al. 2016	Y	Y	N	NA	N	N	N	Y
2	van de Gaar et al. 2016	Y	Y	Y	NA	NA	NA	U	Y
3	Zytnick et al. 2016	Y	Y	N	NA	Y	Y	Y	Y
4	van de Gaar et al. 2017	Y	Y	Y	NA	Y	Y	Y	Y
5	Zahid et al. 2017	Y	Y	U	NA	Y	Y	Y	Y
6	Dallacker et al. 2018	Y	Y	Y	Y	Y	Y	Y	Y
7	Lundeen et al. 2018	Y	Y	U	NA	Y	Y	Y	Y
9	SanGiovanni et al. 2018	Y	Y	U	NA	Y	Y	U	Y
10	Woo Baidal et al. 2018	Y	Y	N	NA	Y	Y	Y	Y
11	Kim et al. 2019	Y	Y	Y	NA	Y	Y	Y	Y
12	Smith et al. 2019	Y	Y	Y	NA	N	N	U	Y
14	Eliason et al. 2020	Y	Y	Y	NA	Y	Y	Y	Y
15	Justiz et al. 2020	Y	Y	U	NA	Y	Y	Y	Y
17	Thomson et al. 2020	Y	Y	Y	NA	Y	Y	Y	Y
18	Carl et al. 2021	Y	Y	U	NA	Y	Y	U	Y
23	Choi et al. 2022	Y	Y	U	NA	Y	Y	U	Y
28	Sylvetsky et al. 2022	Y	Y	U	NA	N	N	U	Y
29	Zhang et al. 2022	Y	Y	U	NA	Y	Y	U	Y
30	Abdel Rahman et al. 2024	Y	Y	Y	NA	Y	Y	Y	Y
31	Talati et al. 2024	Y	Y	U	NA	Y	Y	U	Y
35	Fuster et al. 2025	Y	Y	U	NA	Y	Y	U	Y

Table S3B. JBI qualitative checklist.

No.	Study	Q1	Q2	Q3	Q4	Q5	Q6	Q7	Q8	Q9	Q10
16	Miller et al. 2020	U	Y	Y	Y	Y	N	N	Y	Y	Y
19	Chan et al. 2021	U	Y	Y	Y	Y	N	N	Y	Y	Y

No.	Study	Q1	Q2	Q3	Q4	Q5	Q6	Q7	Q8	Q9	Q10
22	Prada et al. 2021	U	Y	Y	Y	Y	N	N	Y	Y	Y
24	Christensen et al. 2022	U	Y	Y	Y	Y	N	N	Y	Y	Y
25	Fleming-Milici et al. 2022	U	Y	Y	Y	Y	N	N	Y	Y	Y
32	Abdel Rahman et al. 2025	U	Y	Y	Y	Y	Y	Y	Y	Y	Y

Table S3C. JBI quasi-experimental checklist.

No.	Study	Q1	Q2	Q3	Q4	Q5	Q6	Q7	Q8	Q9
13	Bradley et al. 2020	Y	NA	NA	N	Y	N	Y	Y	Y
21	Lee et al. 2021	Y	NA	NA	N	Y	Y	U	Y	Y

Table S3D. JBI cohort checklist.

No.	Study	Q1	Q2	Q3	Q4	Q5	Q6	Q7	Q8	Q9	Q10	Q11
33	Bestle et al. 2025	Y	Y	Y	NA	Y	Y	Y	Y	Y	Y	Y
34	Cui et al. 2025	Y	Y	U	NA	Y	Y	U	Y	Y	Y	Y

Table S3E. Cochrane RoB 2.

No.	Study	D1	D2	D3	D4	D5	Overall
8	Moran and Roberto 2018	SC	L	L	SC	SC	SC
20	Harris and Pomeranz 2021	SC	L	L	L	SC	SC
26	Hall et al. 2022	L	L	L	L	L	L
27	Harris et al. 2022	SC	L	L	H	L	H

Table S3F. Full-text basis for study-level judgements.

No.	Study and tool	Rationale
1	Munsell et al. 2016 — JBI analytical cross-sectional	Online panel and eligibility described; bespoke exposure/outcome attitude scales explicitly reported as not tested for validity or reliability; no multivariable confounding strategy; statistical comparisons otherwise appropriate.
2	van de Gaar et al. 2016 — JBI analytical cross-sectional	Eligibility, setting and paired samples described; trained observers and standardised anthropometry; questionnaire items derived from prior Dutch studies but validation for the parent/child intake outcomes not established; agreement statistics appropriate. Q5-Q6 not applicable to a measurement-agreement objective.
3	Zytnick et al. 2016 — JBI analytical cross-sectional	Eligibility, dyad selection, missing-data exclusions and setting were reported. Exposure/attitude questions were not tested for validity or reliability (Q3=N). The sports-drink consumption outcome was based on validated 2010 NYPANS questions (Q7=Y). Prespecified demographic and physical-activity confounders were addressed using weighted multivariable logistic regression; survey design was incorporated. Q4 was not applicable because no diagnostic condition was assessed.
4	van de Gaar et al. 2017 — JBI analytical cross-sectional	Convenience-school source population and eligibility described; theory-based scales with moderate-to-good internal consistency; prespecified demographic covariates and multivariable regression; parent-reported SSB intake remains susceptible to social-desirability/measurement bias.
5	Zahid et al. 2017 — JBI analytical cross-sectional	Eligibility and Minnesota State Fair convenience setting were explicit. Home-availability and parent-intake measures had prior reliability/validity support, but the study-specific knowledge questionnaire received content review and difficulty testing without further validity testing; therefore Q3=U for the combined exposure set. Child beverage intake used established FFQ items; anthropometry followed standard procedures. Relevant covariates and multivariable logistic models were reported.
6	Dallacker et al. 2018 — JBI analytical cross-sectional	Quota sampling and eligibility of nutritional gatekeepers with children aged 6–12 years were explicit. The computer task was piloted, developed with nutrition/medical expertise and showed acceptable internal consistency

No.	Study and tool	Rationale
		(alpha=0.70). Trained interviewers measured height and weight; child z-BMI used age- and sex-specific thresholds from a representative reference population. Parental education and BMI were prespecified confounders and addressed in adjusted multinomial and linear regression. Cross-sectional temporality remains a limitation but does not change the item-level judgments.
7	Lundeen et al. 2018 — JBI analytical cross-sectional	Sampling, eligibility, response rates, weighting and analytic exclusions were reported. Parent intake used the NHIS SSB screener, but validity/reliability of the single knowledge question was not demonstrated (Q3=U). The adolescent outcome used an FFQ-style screener supported by comparison studies, although frequency rather than volume and possible under/overestimation were acknowledged. Prespecified sociodemographic confounders and survey-weighted multinomial regression were appropriate.
8	Moran and Roberto 2018 — Cochrane RoB 2	Domains D1–D5: some concerns, low, low, some concerns, some concerns; overall some concerns. Electronic random assignment was reported and baseline characteristics were similar, but sequence generation/allocation concealment were not sufficiently detailed in this secondary report (D1). The analysed randomised sample had immediate complete outcomes and no evident deviations (D2-D3). Outcomes were subjective, and participants necessarily knew which labels they saw (D4). This was a secondary analysis without an identified prespecified protocol/SAP and with numerous eligible beverage/outcome/analysis choices (D5).
9	SanGiovanni et al. 2018 — JBI analytical cross-sectional	Clinic eligibility, bilingual criteria, setting and participant characteristics were explicit. The three-item AAP-knowledge measure and beverage-consumption survey were developed through expert review and a four-parent pilot, but formal validity/reliability evidence was not reported (Q3 and Q7=U). Relevant parental and child factors were collected; three candidate predictors were included in multiple linear regression. The small convenience sample and omission of income/food access limit precision and residual-confounding control.
10	Woo Baidal et al. 2018 — JBI analytical cross-sectional	Consecutive WIC recruitment, eligibility, exclusions, bilingual administration and setting were explicit. The principal exposure was a four-item attitude score modified for pregnancy/infancy; the modified items had not undergone formal validation (Q3=N). Parent and infant beverage outcomes used validated quantitative beverage-frequency instruments and serving-size aids (Q7=Y). Literature-informed covariates and appropriate quantile/logistic regression, including an age-restricted sensitivity analysis, were reported.
11	Kim et al. 2019 — JBI analytical cross-sectional	This was primarily an analytical cross-sectional survey, not a pure qualitative study. Eligibility and dental-school clinic setting were described. Sugar-knowledge, intended-control and actual-control instruments underwent expert review, construct testing, internal-consistency/item-discrimination analyses and Rasch assessment. Race, sex, education and income were addressed using generalised linear models. Open-ended barrier responses were a secondary descriptive component.
12	Smith et al. 2019 — JBI analytical cross-sectional	The article explicitly identifies itself as a cross-sectional study; JBI qualitative was inappropriate. Eligibility, three dental-practice settings and sociodemographic exposures were clearly described. The author-developed 28-item oral-interview survey was piloted for comprehension, but no formal validity or reliability assessment was reported for the preference/perception outcomes (Q7=U). Analyses were limited to multiple unadjusted chi-square/cross-tabulation tests; potential confounders were neither prespecified nor controlled (Q5-Q6=N).
13	Bradley et al. 2020 — JBI quasi-experimental	Uncontrolled repeated-measures evaluation of a national campaign. Exposure preceded the post-campaign measurements and dietary intake was measured at five time points with the same validated Intake24 procedure; mixed-effects models accounted for repeated measures and were adjusted for sex and socioeconomic group. There was no concurrent control group and substantial attrition across follow-up, although maximum-likelihood models used all available observations. Q2–Q3 were not applicable to a single-group population campaign evaluation.
14	Eliason et al. 2020 — JBI analytical cross-sectional	Two population survey panels with random-digit telephone sampling, clear analytic eligibility and survey weighting. Parental diet perception was a direct single item used in prior studies; dietary-frequency questions were adapted from BRFSS/NHANES and converted using an NCI algorithm. Child race/ethnicity, maternal education, poverty and WIC/SNAP participation

No.	Study and tool	Rationale
		were prespecified and addressed in survey-weighted, city-cluster-adjusted multivariable models.
15	Justiz et al. 2020 — JBI analytical cross-sectional	Baseline cross-sectional analysis of 685 parent–child dyads from 16 schools in a cluster-randomised trial. Added-sugar recommendation knowledge was assessed with a single survey question without reported validation (Q3=U). Intake was assessed using two unannounced, multiple-pass 24-hour recalls administered by trained staff and processed in NDSR with quality control. Mixed-effects regression incorporated school clustering, robust standard errors and child/parent sociodemographic covariates.
16	Miller et al. 2020 — JBI qualitative	No explicit philosophical perspective was stated (Q1=U). Focus groups, purposive stratification, open-ended facilitation and thematic analysis were congruent with the exploratory aim. Verbatim quotations supported the themes; three researchers reviewed coding and resolved discrepancies by consensus, and saturation was reported. The researcher's cultural/theoretical location and the researcher's influence on the study were not addressed (Q6-Q7=N). Ethics approval and written consent were explicit.
17	Thomson et al. 2020 — JBI analytical cross-sectional	The FLASHE internet-based dyadic study reported eligibility, balanced panel sampling and analytic exclusions. Parenting-practice items were taken or modified from valid and reliable instruments and cognitively tested; added-sugar intake used the FLASHE dietary screener and NCI conversion algorithms. Demographic, BMI, intake and authority covariates were incorporated into the latent-class model; full-information maximum likelihood addressed missing indicators. The absence of dyadic population weights for latent-class analyses limits population inference but does not make the analysis inappropriate.
18	Carl et al. 2021 — JBI analytical cross-sectional	Convenience online panel sampling, purposive Latino oversampling, eligibility and exclusions were explicit. Awareness and discouragement items were adapted from prior studies, professionally translated and grounded in a review of epidemiologic evidence, but study-specific psychometric validity/reliability was not reported (Q3 and Q7=U). A broad set of demographic and behavioural covariates was included in multivariable linear regression. Analyses were explicitly exploratory and not prespecified.
19	Chan et al. 2021 — JBI qualitative	No explicit philosophical positioning; researcher cultural/theoretical location and reflexivity not reported. Ethics approval and consent explicitly reported.
20	Harris and Pomeranz 2021 — Cochrane RoB 2	Domains D1-D5: some concerns, low, low, low, some concerns; overall some concerns for the randomised comparison. The online experiment randomised parents to front-of-package-only versus front-of-package plus nutrition/ingredient information and assessed immediate ingredient-identification accuracy. No important deviations or outcome missingness were evident, and the accuracy outcome was objective. Sequence generation/allocation concealment were not described in sufficient detail in the report, and no prespecified protocol or analysis plan was identified for the multiple product-level outcomes. Cross-sectional descriptive questions were not used to replace the randomised-domain judgement.
21	Lee et al. 2021 — JBI quasi-experimental	Uncontrolled, single-centre, same-visit pre-post evaluation in 149 caregivers. The poster exposure preceded the post-clinic survey; the same knowledge construct was assessed before and after and multivariable logistic regression was reported. There was no control group or repeated pre-intervention measurement. Reliability/validation of the study-specific survey was not established in the report.
22	Prada et al. 2021 — JBI qualitative	No explicit philosophical perspective was stated. Semi-structured interviews, snowball recruitment and largely inductive thematic analysis were congruent with the exploratory question. Two trained interviewers collected data; transcripts were coded using a refined thematic map, with independent duplicate coding of 10% in two rounds and consensus resolution. Findings were illustrated with participant quotations and ethics/consent were reported. Recruitment mainly through research-team contacts was disclosed, but researcher cultural/theoretical positioning and potential influence on data collection/interpretation were not critically examined.
23	Choi et al. 2022 — JBI analytical cross-sectional	Eligibility, national online-panel recruitment, quota sampling, completion flow and setting were explicit. Perceived product attributes and healthfulness were assessed with study-specific Likert-type questions grounded in prior research, but formal validity/reliability was only reported for one derived claim scale; therefore, exposure measurement was unclear overall (Q3=U). Provision frequency was self-reported using a study-specific ordinal measure without

No.	Study and tool	Rationale
		validation evidence (Q7=U). Relevant perceptual, behavioural and sociodemographic variables were included in a partial proportional-odds model selected after testing the parallel-regression assumption.
24	Christensen et al. 2022 — JBI qualitative	No explicit philosophical position was stated. Random sampling from intervention schools, open-ended post-intervention interviews and thematic content analysis were congruent with the evaluative aims. The target sample was justified by saturation; transcripts were coded using iterative independent rounds and reliability testing, with 86% agreement before final coding. Quotations supported the themes and ethics/consent were explicit. The interviewer/analyst role was identified, but cultural/theoretical positioning and reflexive consideration of the prior intervention relationship were not reported.
25	Fleming-Milici et al. 2022 — JBI qualitative	No explicit philosophical perspective was stated. Nine focus groups used an experienced moderator, semi-structured guide and concept sheets appropriate to the study aim. Transcripts were checked, themes developed iteratively by two researchers, a coding manual was used, and independent coders achieved at least 80% agreement; information saturation was reported. Findings were supported by quotations and ethics/consent were reported. Researcher cultural/theoretical location and reflexivity about the information-heavy concept sheets and moderator influence were not addressed.
26	Hall et al. 2022 — Cochrane RoB 2	Domains D1-D5 and overall: low risk. Qualtrics simple randomization allocated participants 1:1:1:1; groups were balanced. The intervention was delivered automatically in a virtual store, the primary product-choice outcome was complete and objectively captured, and no deviations related to trial context were evident. Procedures, hypotheses, primary outcome and analytic plan were preregistered before data collection. The authors transparently reported a reduction in nonessential pairwise comparisons and retained the prespecified primary comparison.
27	Harris et al. 2022 — Cochrane RoB 2	Domains D1-D5: some concerns, low, low, high, low; overall high risk for the principal self-reported attitude/intention outcomes. Random assignment and baseline comparability were reported, but sequence-generation/concealment details were limited (D1=SC). Attrition/exclusions occurred before the final randomised analytic sample and there was no evident differential loss. Outcomes and analyses were prospectively registered. Participants necessarily knew whether they received beverage countermarketing, and the main outcomes were immediate subjective attitudes and intentions that could readily be influenced by awareness of condition (D4=H); this does not negate the experimental effect but increases measurement-bias risk.
28	Sylvetsky et al. 2022 — JBI analytical cross-sectional	Mixed-method descriptive study with separate convenience samples for idea generation and quantitative rating. Eligibility, recruitment changes during COVID-19 and participant characteristics were explicit. The five-minute qualitative prompt generated study-specific beverage characteristics, and the subsequent 50-item influence questionnaire had no reported psychometric validation (Q3/Q7=U). Hispanic-African American comparisons used Mann–Whitney tests with false-discovery-rate correction, but potential demographic and recruitment-mode confounders were neither prespecified nor adjusted (Q5-Q6=N).
29	Zhang et al. 2022 — JBI analytical cross-sectional	Multistage cluster-random sampling, dyad matching, eligibility and exclusions were described. Parent KAP and parent/adolescent free-sugar intake were assessed using questionnaires and a semiquantitative FFQ derived from prior Chinese CDC studies, but study-specific validity/reliability was not demonstrated (Q3/Q7=U). Parent sociodemographic factors and adolescent age, grade and reading style were included as adjustment variables. The proportional-odds assumption was tested, and the adjusted ordinal logistic model was appropriate, although school-level clustering was not explicitly incorporated.
30	Abdel Rahman et al. 2024 — JBI analytical cross-sectional	Multistage stratified proportionate random sampling across 25 Lebanese schools. The TPB-based questionnaire was translated, content-validated, pilot-tested, and showed subscale alpha values above 0.70. Relevant sociodemographic factors and TPB constructs were examined in multivariable regression with model assumptions assessed.
31	Talati et al. 2024 — JBI analytical cross-sectional	Online-panel study of 1,611 Australian parent–child dyads. Eligibility and setting were clear, but several exposure, perception and dietary questions were created for this survey and only a four-dyad read-aloud pilot was

No.	Study and tool	Rationale
		reported; formal psychometric validation was not established. Zero-inflated models incorporated demographic covariates and documented data handling.
32	Abdel Rahman et al. 2025 — JBI qualitative	Purposive sampling across four Lebanese regions, saturation, a twice-piloted topic guide, verbatim transcription/translation, multiple coders, reliability checks and consensus were reported. Research-team reflexivity and cultural positioning were addressed. No explicit philosophical perspective was stated.
33	Bestle et al. 2025 — JBI cohort	Secondary longitudinal mediation analysis within a 3.5-month cluster-randomised trial. Eligibility and cluster allocation were described; parents completed the same questionnaire and seven-day child dietary record at baseline and follow-up. Questionnaire development included translation, cognitive interviews, field testing, CFA and internal consistency assessment; dietary records required at least four valid days. Models adjusted for parental education, child sex and BMI and included participant random effects. Q11 (appropriate statistics)=Y. Outcome-free-at-baseline is not applicable to a change/mediation outcome.
34	Cui et al. 2025 — JBI cohort	Two-wave panel with 4,118 baseline participants and 3,962 retained (96.2%). Parent knowledge used an adapted, widely used six-item quiz without study-specific validation; child SSB volume was self-reported. Confounders were identified and addressed using two-way fixed-effects models with class-clustered robust standard errors. Attrition was quantified and compared. Q11 (appropriate statistics)=Y.
35	Fuster et al. 2025 — JBI analytical cross-sectional	Cross-sectional online survey of 1,006 caregivers with explicit eligibility criteria. The survey was informed by prior work and underwent a 10-person pilot and 20-person soft launch, but formal psychometric validation of the bespoke attitude and 30-day frequency measures was not reported. Multivariable logistic models adjusted for demographics, restaurant use and attitudes.

Note: JBI cross-sectional items: Q1 inclusion criteria; Q2 participants and setting; Q3 exposure measurement; Q4 objective criteria for condition; Q5 confounders identified; Q6 confounding strategy; Q7 outcome measurement; Q8 statistical analysis. JBI qualitative items follow the standard ten questions on congruity, researcher positioning/influence, representation, ethics, and conclusions. JBI quasi-experimental items follow the standard nine questions on temporal clarity, comparison, co-interventions, control, repeated measurement, follow-up, measurement consistency/reliability, and analysis. JBI cohort items follow the standard eleven questions on group similarity, exposure measurement, confounding, outcome status/measurement, follow-up, attrition, and analysis. RoB 2 domains are randomisation, deviations, missing outcomes, outcome measurement, and selective reporting.
